# Home intravenous antibiotic treatment for acute pulmonary exacerbations in cystic fibrosis — Is it good for the patient?

**DOI:** 10.4103/1817-1737.53346

**Published:** 2009

**Authors:** Iara Maria Sequeiros, Nabil A. Jarad

**Affiliations:** *Bristol Adult Cystic Fibrosis Centre, Department of Respiratory Medicine, Bristol Royal Infirmary, United Kingdom*

**Keywords:** Cystic fibrosis, home, treatment

## Abstract

There is a worldwide drive for the home management of chronic respiratory diseases. With the widespread use of home intravenous (IV) treatment for cystic fibrosis (CF) pulmonary exacerbations (PExs), evidence pointing to an inferior outcome of care for home-treated patients in comparison to hospital-treated patients is a cause of concern. Currently, patients who self-administer IV antibiotics at home are provided with equipment and instructions on the use of antibiotics. Policies vary; but in most UK centers, these patients are then followed up by the multidisciplinary team only on days 1, 7 and 14 of the treatment course. We aimed to review the current published literature in search for evidence for the value and the shortfalls of self-administered IV treatment at home for acute PExs in CF patients in comparison to conventional hospital treatment. We searched the electronic database system Medline for published papers regarding studies comparing home- and hospital-based IV antibiotic treatment for both adult and pediatric CF patients. Sixteen studies were identified and grouped into those that showed a similar outcome between home and hospital treatment and those that showed an inferior outcome for home management. Most studies were retrospective or inadequately powered to provide clear answers. Ideally, outcome of care for home treatment should be at least equal to outcome for hospital treatment. Extensive efforts should be made to standardize therapies preserving the advantages of home management and addressing the perceived reasons for an inferior outcome. Until further studies provide definitive answers, treatment at home should be reserved for adequately selected patients and individualized depending on the unique settings of each CF center and specific patients' requirements. There is great need for a prospective randomized controlled trial comparing home and hospital treatments in order to clarify this matter.

Delivery of health care in the UK and Europe is changing. There has been a drive for management of chronic conditions at home. Although not specifically suggested, management of acute exacerbations of clinical conditions such as chronic obstructive pulmonary disease (COPD), bronchiectasis and CF is also frequently being done at home.

In our center, 7 out of every 10 CF patients suffering from PExs are treated at home by self-administered IV antibiotics.[1] The chosen site of treatment, whether at home or in hospital, normally depends on several factors, including severity of the exacerbation, concomitant CF-related complications, patients' own preferences, competence of patients in self-administering IV antibiotics and general home circumstances. The usual practice in most CF centers is that following the decision to start IV antibiotics, the site of management (home or hospital) is chosen after a consensus between the treating CF team and the patient.

Patients who are started on home treatment are provided with the prescribed antibiotics, diluents, syringes, needles and a set of instructions on how to use the drugs given. Monitoring of patients taking home IV antibiotics differs according to the resources and policies of each CF center. In most UK centers, patients are followed up by the multidisciplinary team only on days 1, 7 and 14 of the treatment course.

## Research on Outcome of Care of Patients on IV Antibiotics — Home Versus Hospital

The outcome of care for home versus hospital management of acute PExs has been examined over the years. We searched the electronic database system Medline for published papers regarding studies comparing home- and hospital-based IV antibiotic treatment for both adult and pediatric CF patients.

Studies differed in design, but most were retrospective; and in nearly all of them, common outcome of care measures included change in spirometric values and body weight.

## Studies that Showed Similarity of Outcome

A study published in 1988[2] revealed home IV treatment as having potential advantages over hospital treatment in a cohort of pediatric CF patients. Thirteen patients were carefully selected and trained before home treatment was started. A CF specialist nurse had an essential role in the home treatment system, performing home visits at least once a week, reinforcing compliance and assessing the patient's general condition, including lung function. There were no significant differences in weight and forced expiratory volume in 1 second (FEV_1_) between the home treatment and the hospital treatment at the end of the treatment period, but forced vital capacity (FVC) was better after home treatment. All patients preferred home treatment.

Other studies have been carried out confirming that home IV antibiotic treatment can be safe and effective. Such studies found that the outcomes of care at home and hospital were similar, but these mainly considered cohorts with a small number of patients, single courses of treatment and carefully selected patients (those with good compliance and social support at home). Comparative studies have also shown that home IV antibiotic therapy is cost saving, reducing the economic burden on patients and health care providers.[3–10]

## Studies that Showed Inferior Outcome for Home Management

A number of studies from adult and pediatric CF centers have demonstrated inferior outcomes for home-treated patients when compared to hospital-treated patients in relation to lung function and weight gain.

In a controlled comparison of outcome of home versus hospital therapy,[11] FVC and arterial oxygen tension (PaO_2_) were found to improve to a significantly greater extent in hospital. The authors concluded that these differences were probably explained by differences in pre-treatment values, with hospital-treated patients starting at lower values and consequently having more potential for improvement.

Wolter *et al.*,[12] in a small randomized study reported that patients treated in hospital felt lesser fatigue than home-treated patients. This may reflect the increased activity of patients at home, who continue to carry out daily duties. Hospital patients expressed a greater degree of mastery (feeling of control over the disease and its consequences) with a higher total improvement in quality of life, whilst home-treated patients had lesser disruption in family life and sleep. Although not statistically significant, there was a trend towards overall greater improvement of lung function tests in the hospital-treated group in comparison with the home-treated group. Home-treated patients had significantly fewer investigations performed as compared to inpatients. Home therapy was considered cheaper for families and the hospital. Authors emphasized appropriate patient selection for home therapy to be successful. Patients considered noncompliant were excluded from the study.

Bosworth and Nielson[13] reported outcome of home care with minimal supervision compared with outcome of hospital care and also found that home-based care produced significantly poorer outcomes. The average duration of treatment was twice as long for those treated at home compared with the hospitalized patients, and the time until the next exacerbation needing IV antibiotics was shorter by one third. Home care, as delivered in this study, increased the overall cost of health care by as much as 30%, because of the longer and more frequent courses of IV antibiotics.

A retrospective audit[14] conducted to compare the efficacy of home versus hospital treatment also revealed significantly greater improvements in lung function (as determined by spirometric measures) in the hospital-treated patients.

In 2000, a Cochrane Collaboration Review[15] interested in determining whether home IV antibiotic therapy was as effective as inpatient treatment, selected only 1 randomized trial from the literature.[12] The group recommended the initiation of randomized control trials to compare the 2 approaches.

Thornton *et al.*,[16] examined the long-term clinical outcome of patients receiving IV antibiotics in a large retrospective study on 116 adult CF patients who received 454 courses of IV antibiotics over the course of the study year. At the end of 1 year, there had been a mean percentage decline in FEV_1_ compared with baseline average in home-treated patients, but an improvement in hospital-treated patients. There were statistically significant greater improvements in lung function and nutritional status amongst hospital-treated patients compared with home-treated patients. Differences in outcome were apparent after 1 course of IV antibiotics and were maintained after 1 year of treatment.

In another publication, the same group performed a full cost-effectiveness evaluation comparing home- and hospital-based treatment with IV antibiotics in adults with CF.[17] Treatment was considered effective (as defined by maintenance of baseline average FEV_1_ over the 1-year study period) in a larger number of hospital-based (58.8%) patients as compared to home-based (42.6%) patients. Besides being more effective, hospital treatment was considered to be more expensive than home treatment. Economic analysis demonstrated that the improved clinical effectiveness achieved with hospital-based treatment may only be obtained with home treatment employing considerable extra resources.

A recent North American study on pediatric CF patients[18] examined retrospectively 143 PExs in 50 patients in relation to the location of completion of antibiotic treatment, either at home or in hospital. Treatment of PExs in both groups resulted in significant improvement of lung function, oxygen saturation and weight. Hospital therapy, however, resulted in significantly greater improvement in FEV_1_ and required shorter duration of treatment as compared to home-based therapy.

Another comparative study[19] on CF patients showed a greater improvement in FVC for hospital-treated patients, again suggesting that PExs were not as effectively treated at home. On the other hand, elements of quality of life seemed to be improved when treatment was undertaken at home.

The obvious question is whether patients who were treated in hospital had more severe lung disease than those treated at home. In fact, in most studies, baseline characteristics of patients treated at both the sites did not differ. In other words, it was not the severity of PExs or the degree of the disease that determined the site of treatment.

Despite the importance of this matter, there have been no adequately powered prospective studies comparing the outcome of PExs, probably because of the difficulties in selecting and randomizing patients to the sites of treatment in an independent fashion. As a probable consequence, appropriate assessment of quality of life as a result of treatment at both the sites has not yet been done. Furthermore, residual inflammation, as measured by inflammatory markers at the end of the IV course, and its relation with time until the following exacerbation have not been addressed in any study.

## Possible Reasons for Difference in Outcome

Hospital management is not favored by most CF patients, who prefer home therapy.[6,10,11,17] Hospital treatment is probably disruptive for patients and their families, taking patients away from school activities, work activities and social lives for considerable amounts of time. There are also financial strains on patients due to earning losses as a result of time off from work; and expenses for traveling to hospital, especially if the treatment center/hospital is at a considerable distance from the patient's home. After numerous admissions throughout their lives, patients and their families become acquainted with many aspects of IV drug administration and often want to start self-administration of these medications, avoiding hospital admissions.[2] Reasons for patients' preference for home treatment are outlined in Table 1.

**Table 1:** Reasons why patients prefer home IV antibiotic treatment

Less interruption to education and career
Reduced earning losses and traveling expenses
Improved quality of life
Tastier food
More facilities to exercise
Less disruption to sleep
More convenient timing of drug administration
Reduced risk of cross infection
Lack of hospital beds

CF patients with PExs requiring IV antibiotics place a great strain on the capacity of the hospitals in terms of the available number of beds, and on their manpower and other financial resources with repeated admissions. Accommodation and boarding for patients and, sometimes, members of their families account for the largest fraction of hospital costs for inpatients. Equipment and drugs make up the largest proportion of home therapy costs.[12]

The superior outcome of hospital management over home treatment has been attributed to closer supervision and direct input by the multidisciplinary team, including physiotherapists, dieticians and nursing staff, throughout the period of hospital stay,[10,16] ensuring increased adherence to treatment. Albeit unproven, bed rest during PExs has also been widely regarded as another reason for the favorable outcome of hospital treatment.

Conversely, there are numerous reasons why home treatment could be clinically less effective in treating PExs in CF patients [Table 2]. Considerable commitment is required from patients who are on home-based treatment; as, in addition to their treatment schedules, they have to maintain their domestic routines and social lives, as well as fulfill educational and work commitments. Continuing with normal life and not taking time off work or school would mean maintaining higher general activity levels. These patients are probably not getting the amount of rest they need as a part of their treatment.[17] Self-performed physiotherapy may not be as effective during PExs compared to the treatment provided by a professional physiotherapist, and calorie intake may suffer without daily encouragement.[10]

**Table 2:** Reasons why health care professionals are concerned about the practice of home IV treatment

Reduced medical input
Reduced input from physiotherapists and dieticians
Possible lack of compliance with the IV treatment
Lack of rest
Reliance on patients to diagnose complications

Also, some antibiotic regimens for home treatment are adapted to make administration more convenient and more compatible with work and school hours.[10] This includes twice-daily beta-lactam antibiotics versus the recommended thrice-daily regime. Another important issue is adherence, which is recognized as being potentially poor in CF[20] and may be worse in some patients on home IV treatment. Although assessed by the multidisciplinary team for competency in terms of self-administration of drugs, the level of adherence of patients to treatment is not truly known. This is a widely known phenomenon, often revealed when considerable amounts of unused antibiotics and other drugs are returned by patients and their families to the caring CF center.

## The Way Forward

Ideally, home treatment should be as effective as hospital treatment and clinical improvement not sacrificed on the basis of economic considerations and convenience.[10] It is notable that no published study so far has shown a better outcome for home treatment compared to hospital treatment. Patients should be informed of the outcome of home-based treatment in comparison to the outcome of hospital-based treatment, in order to enable them to make an informed decision about where they would like to be treated.

The factors discussed in the article may collectively make home treatment far from optimal. It is therefore imperative that home treatment, as is presently established, be reviewed and an effort made in order to improve outcome, although this will probably have resource-related implications.[17]

Extensive efforts should be made to standardize therapies received by patients in the home and hospital settings. These measures should address the balance of preserving the advantages of home management vis-à-vis the disadvantages by adequate input from the caring team, sufficient to address the perceived reasons for an inferior outcome. The four key elements of treatment during PExs should be reinforced: adherence to regular IV antibiotics, intensive physiotherapy, increased intake of nutrients and sufficient rest. Potential methods to improve outcome at home should be considered for further prospective research.

The conflict between patients' preference for home treatment and health providers' concern to achieve a favorable outcome of care during stages of clinical instability in CF is ongoing. This is currently handled in variable ways by different CF centers in the UK. Most centers feel that they have to offer some kind of home treatment, although a small number of centers do not.

Others prefer a happy medium of starting treatment in hospital and then discharging patients a few days later to complete the antibiotic course at home. Some CF centers prefer not to treat patients at home for 2 successive PExs.

Finally, until now, most studies on outcome of care have been retrospective and were influenced by patient selection bias. There is considerable need for a prospective, adequately powered randomized controlled research to assess the outcome of care in hospital and at home during PExs, including stratifications in terms of those who benefit from home management and those who do not. Studies should include measurement of CF-related quality of life. There is even greater need to examine whether intense and more frequent assistance of patients by members of the multidisciplinary team during PExs treated at home is a helpful and viable option. Until then, evidence pointing to an inferior outcome of care for home treatment compared to hospital treatment in adult and pediatric CF patients during PExs will continue to be a cause of concern and discomfort to the CF community.
